# Correlation between radiography and motions of foot and ankle during gait using 3D multi-segment foot model

**DOI:** 10.1186/1757-1146-7-S1-A132

**Published:** 2014-04-08

**Authors:** Dong Yeon Lee, Sang Gyo Seo, Ji-Beom Kim, Sung Ju Kim, In Ho Choi

**Affiliations:** 1 Orthopedic Surgery, Seoul National University Hospital, Seoul, South Korea

## Background

The purpose of this study was to provide basic quantitative data to minimize the injury occurring during forward breakfall by the comparative analysis of biomechanical factors through 3D motion analysis, analysis of ground reaction force, and EMG analysis of the forward breakfall of the martial arts targeting 10 skilled and 10 unskilled subjects.

## Methods

In this study, three-dimensional motion analysis, the nine high-speed camera (Motionmaster 100, KOR) was used, the desired total floor reaction force device (ATMI, USA) 2 units was measured using an impact force. And the floor reaction force and three-dimensional motion analysis program was used for the Kwon3dXP. Group differences for verification and program SPSS 21.0 was used.

## Results

The following are the findings. Firstly, the total time taken for forward breakfall of the martial arts showed 1.53±0.04 s for skilled, and 1.41±0.06s for unskilled subjects (*p*<*.01*).

Second, during forward breakfall of the martial arts, the skilled subjects came up with significantly faster impact velocity in the primary point of impact (E2) (*p*<*.001*), but the unskilled subjects showed significantly faster impact velocity in the secondary point of impact (E3) (*p*<*.001*)*.*

Third, the forward breakfall of the martial arts did not show any difference between left and right side in the reaction force, but unskilled subjects proved a significantly greater forward and backward reaction force in the secondary point of impact(E3) both right and left sides(*right: p*<*.01*, *left: p*<*.001*). The skilled subjects showed a significantly greater vertical reaction force in the primary point of impact (E2) (*right: p*<*.001*, *left: p*<*.001*), and unskilled subjects showed a larger vertical reaction force in the secondary point of impact (E3), respectively (*right: p*<*.01*, *left: p*<*.05*)*.*

## Conclusion

Therefore, in order to reduce the impact force when the forward motion action Break fall slowly to reduce the impact velocity and the impact of the hand compared to alleviate elbow seems to be good.

